# Impact of the Composition of Cosmetic Formulations on the Skin Barrier and Film-Forming Potential of a Skin-Protective Biopolymer

**DOI:** 10.1021/acsomega.6c05501

**Published:** 2026-08-25

**Authors:** Leticia Kakuda, Rafaela A. Zito, Patricia M. B. G. Maia Campos

**Affiliations:** School of Pharmaceutical Sciences of Ribeirão Preto, University of São Paulo, Ribeirão Preto, Sao Paulo 14040-903, Brazil

## Abstract

Air pollution-associated skin damage has increased interest in cosmetic strategies that support skin barrier function. However, the performance of film-forming properties biopolymers can be influenced by cosmetic formulation composition. In this context, this study aimed to develop and characterize gel and gel-cream formulations with or without 1% of tara and red algae-derived biopolymer. These formulations were evaluated for rheological, texture profile, sensory properties, and short-term clinical efficacy. All formulations remained stable for 28 days, with pH values between 5.0 and 5.5. The addition of biopolymer in the formulations was associated with lower consistency index values, from 79.6 ± 1.4 to 46.9 ± 0.7 in gels and from 192.1 ± 1.1 to 156.1 ± 0.9 in gel-creams. Firmness, consistency, and viscosity index were also lower in the formations added with biopolymer. The work of shear parameter was not significantly changed and biopolymer did not alter the sensory properties of the formulations. In the short term clinical efficacy study, significant increases in stratum corneum water content and reductions in transepidermal water loss were observed at 1 h for the gel containing the biopolymer and at 2 h for the gel-cream containing the biopolymer (*p* < 0.05). At 2 h, both formulations significantly improved smoothness, roughness, and scaliness parameters of skin microrelief compared to baseline values, and the gel-cream containing the biopolymer significantly improved smoothness when compared to its vehicle (*p* < 0.05). Overall, the gel showed an earlier skin barrier-related response, whereas the gel-cream showed the clearest vehicle-controlled effect on skin smoothness. Film formation was inferred indirectly from rheological and skin biophysical findings. Direct film characterization and longer-term studies are required to confirm the proposed mechanism and persistence of the effects.

## Graphical abstract

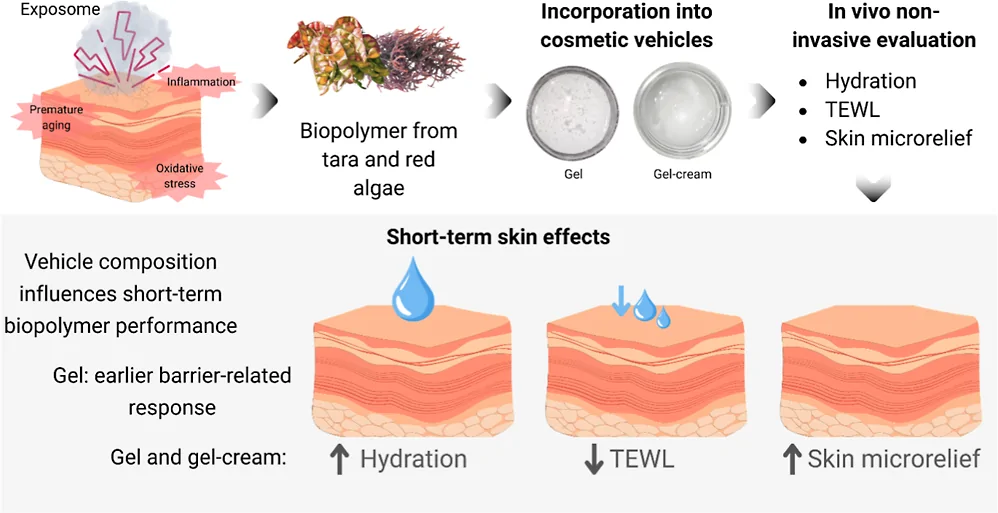

## 1 Introduction

The exposome comprises multiple external and internal factors that influence skin health, including UV radiation, sleep cycle, diet, temperature fluctuations, humidity, and pollutants.^1,2^ Air pollution is particularly relevant because particulate and gaseous pollutants can interact with the skin surface, increase reactive oxygen species production, promote inflammation, and contribute to premature aging and barrier impairment.^2–6^

Cosmetic products intended for use in polluted environments have received increasing attention.^2,7–9^ In this context, skin-protective formulations may be designed to reinforce the skin barrier and reduce the interaction between the skin surface and external aggressors. One strategy involves the formation of a nonocclusive layer on the skin surface.^6,10^ The performance of this layer depends not only on the film-forming ingredient, but also on its interaction with the other components of the formulation, which can influence spreading, deposition, drying, and residence on the skin.^9^

Film-forming cosmetic ingredients may be synthetic or naturally derived. Polysaccharide-based biopolymers are of interest because molecular mass, branching, charge, hydration, and interchain interactions can influence the cohesion of a deposited layer.^11^ These polysaccharides contain hydrophilic groups that can interact with water molecules and may contribute to the formation of a hydrated layer on the skin surface. Polysaccharide-containing topical formulations have been associated with skin hydration and skin-surface effects.^6,12–14^

Previous studies have evaluated film-forming cosmetic systems and the clinical responses of polysaccharide-containing formulations.^15,16^ However, the influence of cosmetic formulation composition on the performance of the same film-forming biopolymer remains less explored. The scientific question addressed in the present study is whether the same concentration of tara/red algae biopolymer behaves differently when added to gel and gel-cream formulations. By maintaining the biopolymer concentration and changing the cosmetic formulation, the influence of formulation composition to rheology, texture profile, sensory properties, and short-term skin effects can be evaluated.

Gels are mainly formed by an aqueous polymeric network, whereas gel-creams include an oil phase and an emulsifying system.^17^ These compositional differences may alter water distribution, polymer hydration, rheological organization, spreading behavior, evaporation, and interaction with the skin surface.^12,13^ Therefore, we hypothesized that changing the formulation from gel to gel-cream would influence the physical–mechanical properties of the formulations and their short-term effects on stratum corneum water content, transepidermal water loss, and skin microrelief.

In this context, noninvasive in vivo skin biophysical and imaging techniques provide valuable insights into how cosmetic formulations interact with the skin. These methodologies enable the measurement of key parameters, such as transepidermal water loss, skin hydration, and microrelief, allowing a comprehensive evaluation of how formulation composition influences both physicochemical properties and short-term barrier-related performance.^15^

Therefore, this study aimed to develop and characterize gel and gel-cream formulations containing tara/red algae biopolymer, focusing on the impact of the cosmetic formulation on rheological behavior, sensory properties, texture, spreadability, and short-term skin barrier-related performance. The integrated evaluation of the same biopolymer concentration in two different cosmetic formulations may contribute to a better understanding of how formulation composition modulates the performance of film-forming biopolymers and may support the development of skin-protective cosmetic formulations.

## 2 Methods

### 2.1 Development of the Formulations and Preliminary Stability Tests

Gel and gel-cream formulations containing 1% of the biopolymer, as well as the corresponding formulations, were developed as described in Table 1. The developed formulations were submitted to stability tests. The organoleptic characteristics, that is, the appearance, color, odor, and homogeneity, were examined by visual inspection.^16^ The pH analysis was performed with a digital pH meter equipped with an electrode for semisolid samples. Phase separation analysis was evaluated after three cycles of centrifugation at 3000 rpm for 30 min (Fanem, São Paulo, Brazil). The samples were stored at room temperature (25.0 ± 2.0 °C) and were submitted to thermal stress at 37.0 ± 2.0 °C and 45.0 ± 2.0 °C.^18^ The measurements were performed 24 h, 7, 14, 21, and 28 days after the preparation of the formulations, in triplicate.^6^

**Table 1:** Composition of the Formulations (w/w %)^a^ ^b^

ingredients (INCI Name)	G1	G2	GC1	GC2
	%	%	%	%
Carbomer	0.50	0.50	0.50	0.50
Disodium EDTA	0.05	0.05	0.05	0.05
Butyl Hydroxy Toluene	-	-	0.02	0.02
Butylene Glycol	3	3	3	3
Behenyl Alcohol, Polyglyceryl-10 Pentastearate, Sodium Stearoyl Lactylate	-	-	1.50	1.50
Glycerin	5	5	5	5
Parfum	0.40	0.40	0.40	0.40
C12–15 Alkyl Benzoate	-	-	4	4
Water	q.s. 100	q.s. 100	q.s. 100	q.s. 100
Kappaphycus alvarezii Extract & Caesalpinia spinosa Fruit Extract	-	1	-	1

a International Nomenclature of Cosmetic Ingredients.

b G1-gel, G2-gel containing 1% of biopolymer, GC1-gel-cream, and GC2-gel-cream containing 1% of biopolymer.

### 2.2 Rheological Behavior

Rheological behavior was evaluated using a DV3T cone-and-plate rheometer (Brookfield, USA) equipped with spindle CP-51. The rotation speed was increased from 0 to 20 rpm with an interval of 2 s for each speed increment, forming an ascending curve composed of the axes “shear rate” versus “shear stress”.^18,19^ The procedure was then reversed to obtain the descending curve. The obtained rheograms allow the observation of the immediate behavior of the formulations after deformation and monitor their evolution during the shear time.

The curves of the rheograms were adjusted according to the Ostwald model to obtain the flow index and consistency index (Pa·s^n^). The minimum apparent viscosity (Pa·s) was obtained from the highest point on the curve.^20^ Samples were stored at 25.0 ± 2.0 °C, 37.0 ± 2.0 °C, and 45.0 ± 2.0 °C, and the measurements were performed after 24 h, 7, 14, 21, and 28 days of preparation, in triplicate.

### 2.3 Texture and Spreadability Profile

Texture and spreadability were evaluated using a TA.XTplus Texture Analyzer (Stable Micro Systems, United Kingdom) with Exponent software. Analyses were performed 24 h after formulation preparation at room temperature.^6,18,21^

The spreadability analysis was performed with the TTC HDP/SR probe, according to the following parameters: trigger force of 15 g, return distance of 25 mm and return speed of 20 mm/s^19^ The amount of formulation used in this analysis was between 35 and 40 g. In this test, the work of the shear parameter was evaluated from the positive area under the curve.^19^

To evaluate the index of viscosity, firmness, and consistency parameters, an A/BE probe of 40 mm was used.^19,20^ The conditions of the test were: contact force of 30 g and return distance and speed were 100 mm and 20 mm/s, respectively.^18,19^ The container was filled to 70% of its capacity. All measurements were performed in quintuplicate.

### 2.4 Sensory Analysis

The study was carried out after approval by the Ethical Committee of the FCFRP/CEP (CAAE: 58730816.5.0000.5403). Formulations were coded and randomly allocated to the application sites. Participants and investigators were blinded to whether each formulation contained the biopolymer or corresponded to its respective vehicle control. However, because gel and gel-cream formulations presented different visual characteristics, blinding regarding the formulation type itself was not fully feasible. Sensory properties were evaluated using the CATA questionnaire (Check All That Apply) questionnaire, which consists of several multiple-choice questions, where the participants marked all parameters that best characterize the formulation in their opinion.^22^ Thirty-two women aged 20–30 years completed the CATA questionnaire immediately after formulations’ application and again 5 min after application. The analyzed parameters immediately after the application were: oiliness, ease of spreading, easy absorption, and firmness. After 5 min of application, the parameters were: oily residue, soft skin, stickiness, hydration, and dry touch.^6^

### 2.5 Clinical Study—Short-Term Effects Evaluation

The short-term clinical efficacy study was conducted in accordance with the Declaration of Helsinki and after approval by the Ethics Committee of FCFRP/USP (CAAE: 58730816.5.0000.5403). Eight healthy women aged 20–25 years with Fitzpatrick skin phototypes II-III were recruited for the clinical study.^23^ All participants were informed about the study procedure and signed an informed consent form. A randomized, vehicle-controlled, intraindividual design was used. Formulations were coded and allocated randomly to the application sites. However, complete blinding of participants and investigators could not be guaranteed because the gel and gel-cream differed in appearance and sensory properties.

The participants had their malar region delimited into four regions with 16 cm^2^, two on each side of the face, where 32 μL of the formulations were applied randomly. The tests started after 20 min of acclimatization in a room with controlled temperature and relative humidity, ranging from 20 to 22 °C and 45 to 55%, respectively.

To evaluate the clinical effects, instrumental measurements were performed using validated biophysical techniques, including transepidermal water loss (TEWL), stratum corneum water content, and skin microrelief analysis. All measurements were conducted in triplicate. The evaluation protocol consisted of baseline measurements (T0) followed by assessments at 1 h (T1) and 2 h (T2) after formulation application. The entire methodology employed noninvasive procedures following established protocols.^20^

The equipment Corneometer CM 825 (Courage & Khazaka, Germany) was used to determine the hydration level of the stratum corneum. This equipment is based on the dielectric constant of water, and capacitance values are measured according to skin water content.^23^ The results are given in arbitrary units (AU), where 1 AU is estimated to correspond to 0.2–0.9 mg of water per gram of stratum corneum.^15^

The skin barrier function was evaluated by TEWL using the Tewameter equipment (Courage & Khazaka, Germany). The method is based on the Adolf Fick principle of diffusion, in which the equipment can measure the water evaporation rate from the skin surface.^6,23^ The results are given in g/(m^2^·h). For each analysis, the probe remained for 2 min on the malar region of the face, and the average of the three measurements was used^15^

Skin microrelief was evaluated using a Visioscan VC 98 (Courage & Khazaka, Germany), which captures high-resolution grayscale images of the skin surface. The Surface Evaluation of the Living Skin software was used to calculate the skin smoothness (SEsm), roughness (SEr), and scaliness (SEsc) indices.^15,23^

### 2.6 Statistical Analysis

Data normality was assessed using the Shapiro–Wilk test. Parametric data were analyzed by one-way or two-way analysis of variance (ANOVA), followed by Tukey’s posthoc test, while nonparametric data were analyzed by the Kruskal–Wallis test with Dunn’s posthoc test. Results are expressed as mean ± standard deviation (SD). All analyses were performed using GraphPad Prism 8 (GraphPad Software Inc., USA) and Origin Pro 8 (OriginLab Corporation, USA), at a significance level of 5% (*p* < 0.05).

## 3 Results and Discussion

### 3.1 Development of the Formulations and Preliminary Stability Tests

The formulations did not show phase separation after centrifugation, and no changes in appearance, color, odor, or homogeneity were observed during the preliminary stability study. These results indicate that the selected ingredients were compatible under the tested conditions and that the incorporation of 1% tara/red algae biopolymer did not promote visible physical instability in either the gel or gel-cream system. This preliminary stability is an important requirement for topical formulations, since changes in phase behavior or organoleptic characteristics may affect product performance, sensory perception, and consumer acceptance.^16,18^

The pH values remained between 5.0 and 5.5 after 28 days of storage, including under thermal stress. This range is compatible with the mildly acidic pH of the skin surface (4.1 to 5.8) and is relevant for maintaining skin barrier homeostasis and formulation tolerability.^6,24^ The maintenance of pH under the evaluated conditions also suggests that the formulation components and the biopolymer did not induce relevant acid–base instability during the study period.

### 3.2 Rheological Behavior

According to the rheograms shown in Figure 1, the four formulations maintained similar rheological profiles, with no peak formation or relevant changes during the 28 day study at the evaluated storage temperatures. All formulations exhibited flow indices below 1, confirming non-Newtonian pseudoplastic behavior. This shear-thinning profile is desirable for topical semisolid formulations because viscosity decreases as shear rate increases, facilitating product spreading during application.^21^

**Figure 1:**
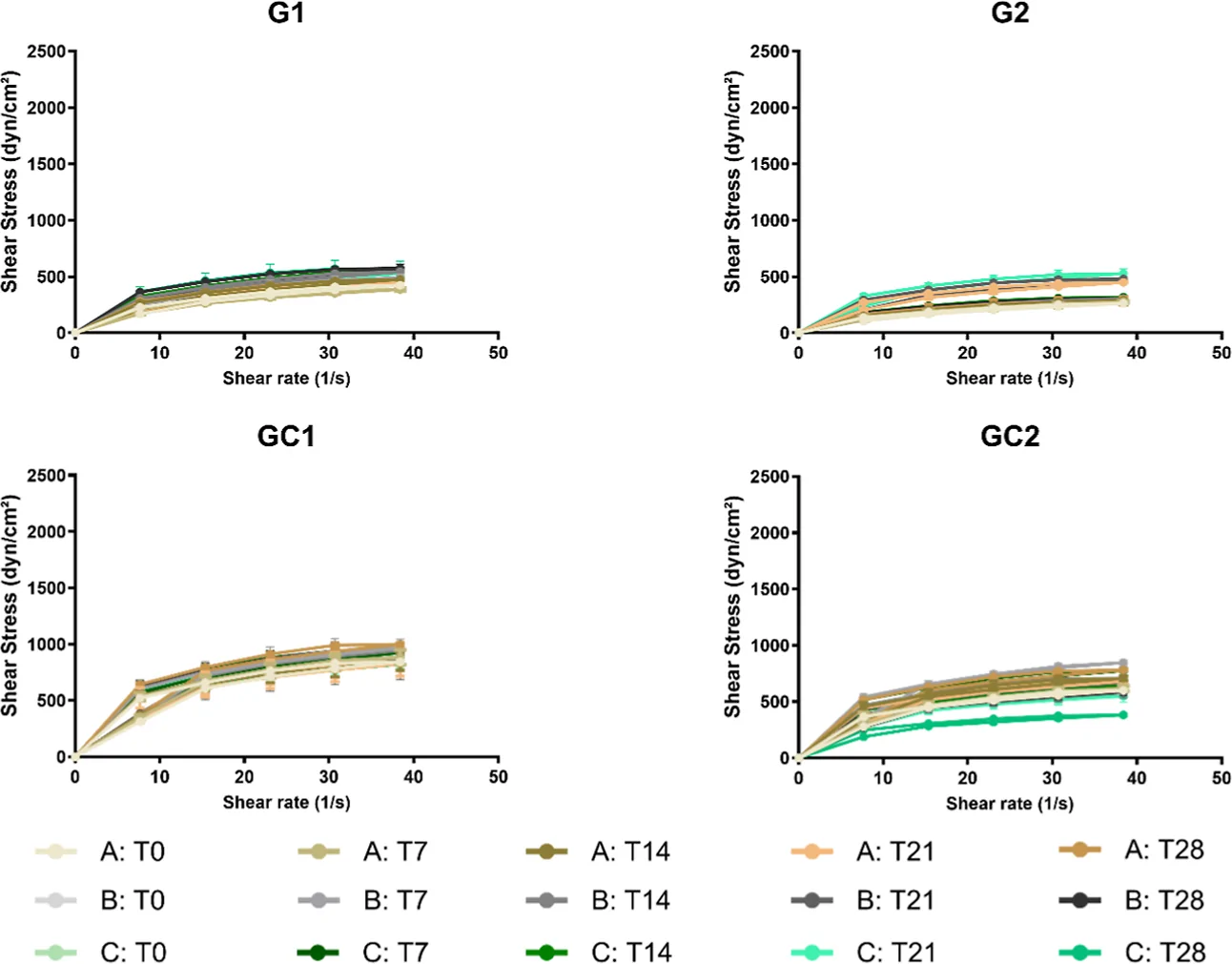
Rheograms of the gel (G1), gel containing 1% biopolymer (G2), gel-cream (GC1), and gel-cream containing 1% biopolymer (GC2), obtained 24 h after preparation (T0) and after 7 (T7), 14 (T14), 21 (T21), and 28 (T28) days of storage at 25.0 ± 2.0 °C (A), 37.0 ± 2.0 °C (B), and 45.0 ± 2.0 °C (C).

The pseudoplastic behavior observed in all formulations is consistent with the presence of carbomer, which forms a water-swollen microgel network and exhibits shear-thinning behavior in topical gel systems.^25,26^ In G2 and GC2, the tara/red algae biopolymer may have further modified this network. This biopolymer is composed of galactomannans from *Caesalpinia spinosa* and sulfated galactans from *Kappaphycus alvarezii*. These polysaccharides contain hydroxyl and sulfate groups that interact with water and may establish intermolecular associations, including hydrogen bonding, thereby contributing to the organization of a hydrated polymeric network. Under shear, partial chain orientation and disruption of these interactions may reduce resistance to flow and contribute to the observed shear-thinning behavior.^27,28^

The area enclosed by the ascending and descending flow curves, known as the hysteresis area, is an empirical measure of the time-dependent structural response of the formulation under the applied shear protocol.^26,29^ A smaller hysteresis area indicates a smaller difference between the two curves and, therefore, less pronounced thixotropic behavior under the measurement conditions.^6,30^ However, this parameter alone cannot determine whether the smaller area results from limited structural breakdown, faster or more complete rebuilding, or a combination of these processes.

Gel-cream formulations exhibited larger hysteresis areas compared with gel formulations, indicating a more pronounced time-dependent structural response under the applied shear protocol (Figure 2). The addition of the biopolymer significantly (*p* < 0.05) reduced the hysteresis area, particularly in the gel formulation. A larger numerical reduction was observed in the gel system. This response suggests that the biopolymer modified the internal organization and the structural breakdown-rebuilding balance of the formulations during shear.^20,29^ However, the hysteresis area should be interpreted as an indirect rheological indicator of thixotropic behavior and not as direct evidence of film formation (see Figure 3).

**Figure 2:**
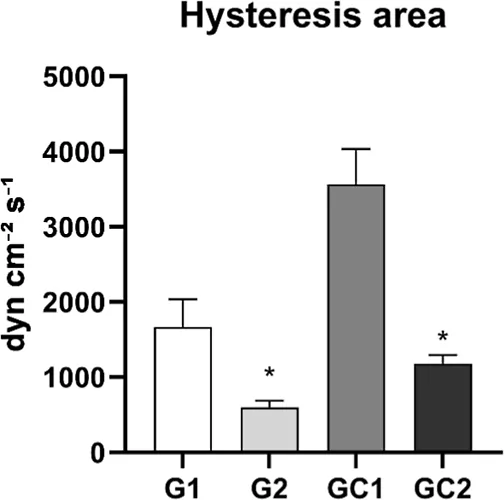
Hysteresis area of the gel (G1), gel containing 1% biopolymer (G2), gel-cream (GC1), and gel-cream containing 1% biopolymer (GC2), evaluated 24 h after formulation preparation at 25.0 ± 2.0 °C. *Significant difference from the corresponding formulation (*p* < 0.05).

**Figure 3:**
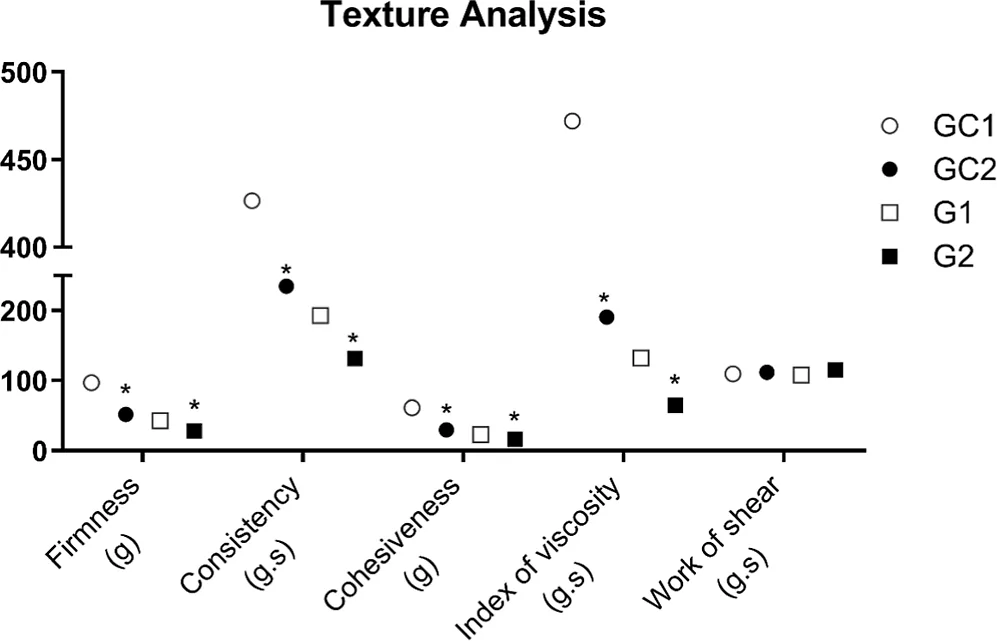
Texture parameters of the gel (G1), gel containing 1% biopolymer (G2), gel-cream (GC1), and gel-cream containing 1% biopolymer (GC2), evaluated 24 h after formulation preparation at 25.0 ± 2.0 °C. Data are presented as mean ± standard deviation, *n* = 5. *Significant difference from the corresponding vehicle formulation, *p* < 0.05.

The smaller hysteresis area of G2 indicates less pronounced time-dependent restructuring within the measurement time scale. Although this behavior may influence formulation performance after application, the rheological measurements do not demonstrate the formation or properties of a deposited film. The larger hysteresis areas of the gel-creams may reflect the combined influence of the oil phase, emulsifying system, carbomer network, and overall emulsion microstructure. Because the gel and gel-cream differed in several formulation components, the contribution of each component cannot be isolated in the present experimental design.^19^

Biopolymers of high molecular weight, above 100 kDa,^31^ may produce a lifting effect by forming a film on the skin surface, resulting in an immediate tensor effect. The studied biopolymer is an interpenetrating polymer network (IPN), meaning it consists of two networks cross-linked in the presence of one another.^32^ One network is composed of galactomannans from tara and the other of sulfated galactans from red algae, both being high molecular weight polysaccharides, with molecular weights around 2300 kDa for galactomannans and 150 kDa for sulfated galactans.^31,33^ Given these characteristics, the studied biopolymer may contribute to the formation of a hydrated polymeric layer on the skin surface, which could be associated with skin-tensing and microrelief effects after application.^34^ This effect may be more evident in gel formulations, which can favor closer contact with the skin surface and may contribute to a second skin-like sensory perception.^14^

Taken together, the hysteresis results indicate that the gel formulations showed a less pronounced time-dependent rheological response than the gel-creams under the applied protocol. This behavior may influence formulation performance after application; however, it should not be interpreted as direct evidence of faster structural recovery or film formation.

The flow index is also associated with spreadability and application behavior.^19^ Among the evaluated formulations, GC2 showed the lowest numerical flow index (0.4 ± 0.1), indicating a numerically more pronounced shear-thinning profile.^21^ However, this difference was not statistically significant (*p* > 0.05), indicating that the flow index was not significantly changed by biopolymer incorporation.

The consistency index reflects the structural consistency of the formulation and is also associated with the firmness parameter obtained in the texture analysis.^19,21^ The incorporation of the biopolymer decreased the consistency index in both formulation types relative to their respective vehicles, indicating reduced structural consistency after biopolymer addition. At the initial point, G1 (79.6 ± 1.4) and GC1 (192.1 ± 1.1) showed higher values than G2 (46.9 ± 0.7) and GC2 (156.1 ± 0.9), respectively, showing that the formulations had higher numerical consistency indices.

Although polymer addition is frequently associated with increased viscosity, the magnitude and direction of the rheological response in multicomponent formulations depend on polymer concentration, hydration, solvent composition, processing conditions, and interactions with other structuring agents. Tara galactomannans have a high affinity for water, and their rheological properties are influenced by concentration and medium conditions.^35^ Carbomer forms water-swollen microgel particles that can organize into a percolated network, and its rheological behavior is influenced by solvent composition, polymer–solvent interactions, and dispersion conditions.^25,26,36,37^ Therefore, the lower consistency index and minimum apparent viscosity observed after biopolymer incorporation may reflect changes in polymer hydration and in polymer–solvent or polymer–polymer interactions within the mixed system. However, because water distribution and polymer-network microstructure were not directly characterized, competition for water and weakening of the carbomer network should be considered possible mechanisms rather than established explanations.

Significant differences were detected only in selected between-formulation comparisons, whereas no significant time-dependent or temperature-dependent changes were observed within the same formulation (*p* > 0.05). These findings indicate that the consistency index remained stable under the evaluated storage conditions. The lower instrumental consistency values were also not influenced sensory analysis responses, indicating that biopolymer did not alter the sensory properties of the formulations.

Minimum apparent viscosity is a rheological parameter associated with formulation flow behavior under shear and may contribute to spreadability and application performance.^21^ No significant temperature-dependent or time-dependent changes (*p* > 0.05) were observed within the same formulation, even under thermal stress, indicating that the minimum apparent viscosity remained stable throughout the study period. G2 showed numerically lower minimum apparent viscosity than G1 across the evaluated storage conditions, and a similar numerical pattern was observed for GC2 relative to GC1. This trend may indicate lower resistance to flow under the applied shear conditions. However, because matched comparisons did not consistently reach statistical significance, the results should not be presented as demonstrating a uniform reduction in minimum apparent viscosity caused by biopolymer incorporation. The absence of time- and temperature-dependent changes nevertheless supports the rheological stability of the formulations under the evaluated conditions.

### 3.3 Texture Profile

Biopolymer incorporation significantly reduced firmness, consistency, and viscosity index relative to the corresponding vehicle formulations (*p* < 0.05), whereas work of shear was not significantly changed (*p* > 0.05). This pattern is consistent with a modification of the mechanical response of the gel and gel-cream networks in the presence of the polysaccharides.^38^ However, because formulation microstructure was not directly characterized, these results should not be interpreted as direct evidence of a specific structural rearrangement.

According to the literature, the incorporation of polysaccharides can modify formulation texture, although the magnitude and direction of the response depend on polymer structure, concentration, hydration, and interactions with other formulation ingredients.^38,39^ Consistent with the rheological findings, altered water distribution and polymer–polymer or polymer–solvent interactions may have reduced the mechanical cohesion of the mixed polymer network. Nevertheless, this explanation remains hypothetical because water distribution and polymer-network organization were not directly evaluated.

Work of shear is an instrumental measure of the energy required to spread a formulation.^40^ Under the applied test conditions, lower values are generally associated with easier instrumental spreading.^19,21^ No significant differences were observed among the formulations for this parameter (*p* > 0.05), indicating that biopolymer incorporation did not significantly modify instrumental spreadability. This result was consistent with the sensory evaluation, in which the formulations were generally perceived as easy to spread.

Firmness, consistency, and viscosity index describe the resistance of the formulation to deformation and flow during the texture analysis. These parameters were lower for G2 and GC2 than for their corresponding vehicles, indicating lower mechanical resistance after biopolymer incorporation. However, these reductions should not be directly interpreted as improved spreadability because work of shear remained unchanged.

Gel-cream formulations exhibited higher texture values than gel formulations. This difference may reflect the combined contribution of the dispersed oil phase, emulsifying system, carbomer network, and overall emulsion architecture. Dubuisson et al. (2018)^41^ showed that oil-phase content and polymer concentration can influence the consistency and sensory properties of cosmetic emulsions. However, because the gel and gel-cream formulations differed in several components, the contribution of the oil phase cannot be isolated in the present study.

Texture analysis is a useful tool in cosmetic formulation development because it enables the instrumental characterization of mechanical properties and their comparison with sensory attributes and application behavior.^19,21,42,43^ Instrumental and sensory measurements may provide complementary information about topical formulations, although direct correspondence should not be assumed for every parameter.

In the present study, G2 showed lower instrumental firmness, consistency, and viscosity index, and the firmness descriptor was selected less frequently for this formulation, without a significant change in work of shear. These results indicate that the biopolymer modified the mechanical properties of the gel without impairing its application-related sensory properties.

### 3.4 Sensory Analysis

Sensory properties are important in cosmetic formulation development because they influence how products are perceived during and after application. These perceptions may be affected by formulation composition and the physical–mechanical properties of the formulation.^19,40^

Immediately after application, the firmness descriptor was selected more frequently for GC1 and GC2 than for the gel formulations, with the lowest frequency observed for G2. A positive relationship between instrumental and sensory properties has been reported.^19^ Accordingly, the more frequent selection of firmness for the gel-creams was consistent with their higher instrumental firmness and rheological consistency index values.

Regarding oiliness perception, the oiliness descriptor was selected less frequently for G1 and G2 than for the gel-cream formulations. This vehicle-dependent profile is consistent with the presence of an oil phase and emollient ingredients in GC1 and GC2, although the contribution of individual components cannot be isolated because the gel and gel-cream differed in several formulation variables. Emollient type and emulsion composition have been reported to influence rheological and textural properties, spreading behavior, and sensory attributes of cosmetic formulations.^40,41,43^

Ease of spreading and easy absorption were frequently selected for both the vehicle formulations and biopolymer-containing formulations, whereas stickiness was selected infrequently (Figure 4). Therefore, biopolymer incorporation did not appear to impair perceived ease of spreading or absorption or to introduce an undesirable sticky after-feel under the evaluated conditions.^14^ These findings describe the participants’ sensory perceptions and do not constitute direct evidence of formulation absorption or film formation.

**Figure 4:**
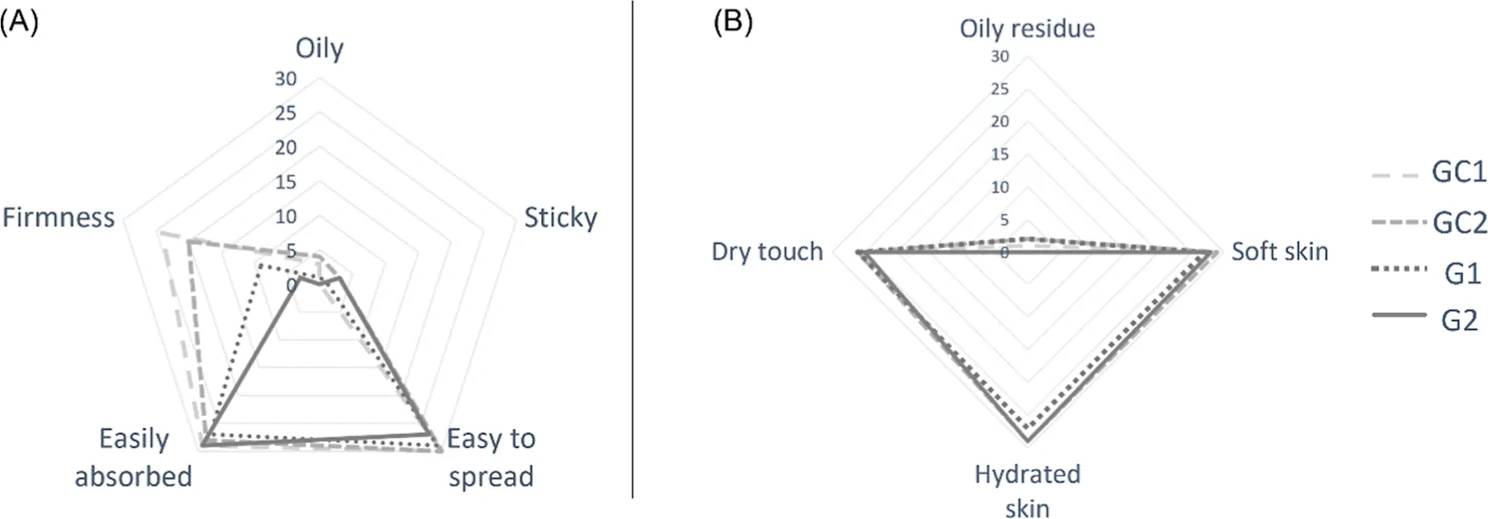
Frequency of sensory properties selected for the gel (G1), gel containing 1% biopolymer (G2), gel-cream (GC1), and gel-cream containing 1% biopolymer (GC2), immediately after application (A) and 5 min after application (B). Data represent the number of participants who selected each descriptor in the CATA questionnaire (*n* = 32).

After 5 min, soft skin, hydrated skin, and dry touch were frequently selected for the evaluated formulations. These descriptors represent the participants’ sensory perceptions and should not be interpreted as instrumental evidence of skin hydration or formulation absorption. The oily residue descriptor was selected more frequently for GC1 and GC2 than for the gel formulations, whereas G2 showed the lowest descriptive frequency of residual oiliness. This pattern is consistent with differences in formulation composition, particularly the presence of an oil phase in the gel-creams, but the individual contribution of each ingredient was not determined.

Overall, the sensory results indicate that formulation composition influenced the perception of firmness, oiliness, and residual oiliness. Although biopolymer incorporation modified the rheological and textural properties of the formulations, it did not produce an evident increase in stickiness or reduce the perceived ease of spreading and absorption.

### 3.5 Clinical Study—Short-Term Effects Evaluation

Stratum corneum water content is an important indicator of skin hydration and can be influenced by individual characteristics, environmental conditions, and topical product application. In the present study, it was evaluated by capacitance measurements, which reflect changes in the dielectric properties of the skin associated with its water content.^44^

At T2, G2, GC1, and GC2 showed significant increases in stratum corneum water content relative to their respective baseline values (*p* < 0.05), whereas no significant change was observed for G1 (Figure 5). The response observed for GC1 indicates that the gel-cream itself contributed to the increase in stratum corneum water content.^45^ This effect may reflect the combined contribution of glycerin, the oil phase, emollients, and the emulsifying system. However, because these components were not evaluated individually, the hydration response should be attributed to the gel-cream as a whole rather than to a specific formulation component^45,46^

**Figure 5:**
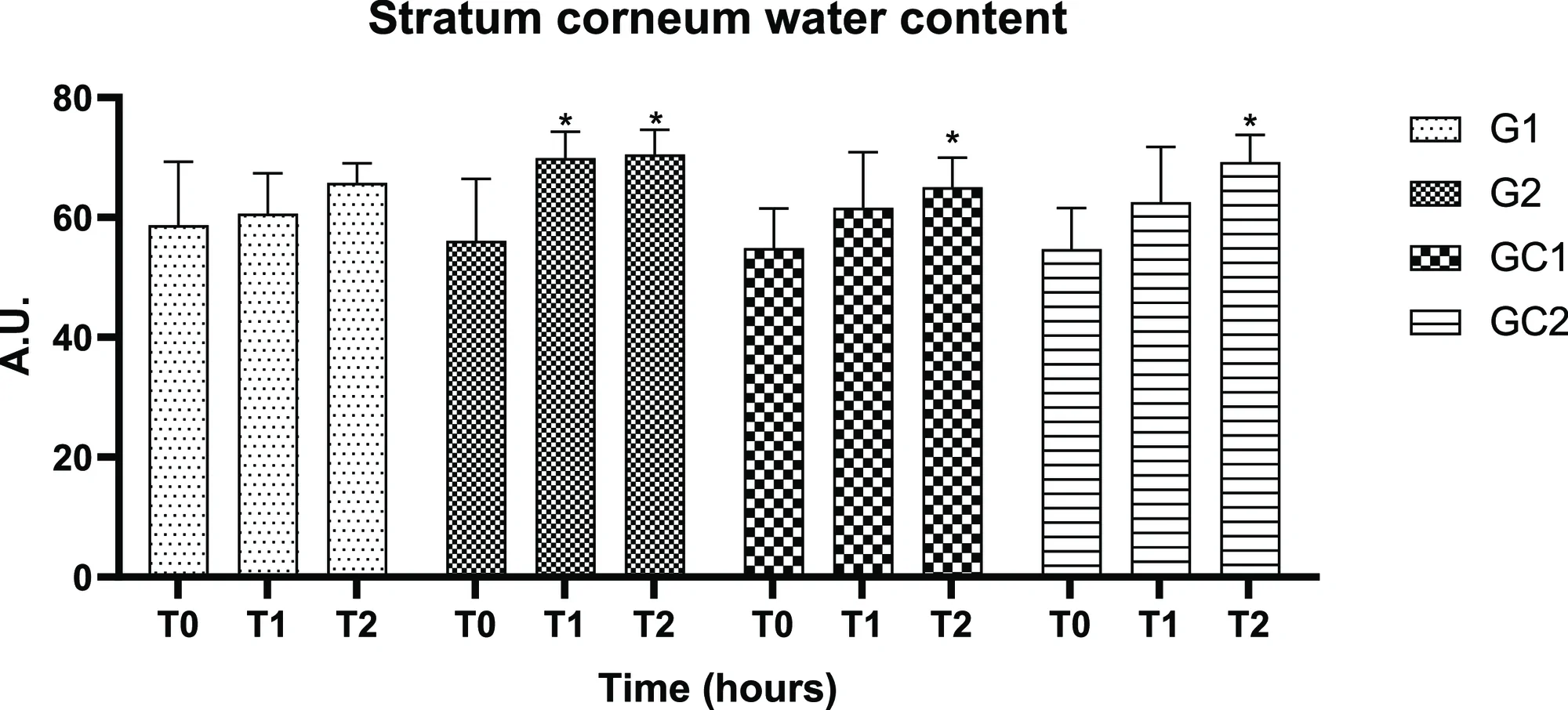
Stratum corneum water content before application (T0) and 1 h (T1) and 2 h (T2) after application of the gel (G1), gel containing 1% biopolymer (G2), gel-cream (GC1), and gel-cream containing 1% biopolymer (GC2). Data are presented as mean ± standard deviation, *n* = 8. *Significant difference from the respective baseline value (T0), *p* < 0.05.

G2 showed a significant increase from baseline at T1, whereas GC2 showed a significant increase at T2 (*p* < 0.05). This temporal pattern indicates an earlier hydration response for the biopolymer-containing gel under the evaluated conditions. Although G2 and GC2 did not differ significantly at the evaluated time points (*p* > 0.05), the earlier significant response observed for G2 suggests that formulation composition influenced the time at which the hydration effect became detectable.

The significant increase in stratum corneum water content observed for G2, together with the absence of a significant change for G1, is consistent with a contribution of the biopolymer to the hydration response of the gel formulation. Furthermore, G2 simultaneously increased stratum corneum water content and reduced TEWL at T1. The concurrent improvement in these two complementary parameters indicates an earlier barrier-related response for the biopolymer-containing gel, consistent with increased water retention at the skin surface.^15^

Galactomannans and sulfated galactans are hydrophilic polysaccharides containing functional groups capable of interacting with water. In particular, the hydroxyl-rich structure of tara galactomannans favors water binding and the formation of hydrated polymeric networks. Therefore, the tara/red algae biopolymer may have contributed to water retention at the skin surface, resulting in the increased stratum corneum water content observed after application.^11,35,47^ The earlier response observed for G2 can also reflect differences in formulation composition, including the absence of an oil phase and emulsifying system in the gel. In the sensory analysis, the gel formulations were perceived as easily absorbed; however, this descriptor represents participant perception rather than a direct measurement of formulation absorption.

In addition to direct water binding, the deposition of a hydrated polymer-rich layer may contribute to reduced water evaporation from the skin surface. Thus, the combined increase in stratum corneum water content and reduction in TEWL observed for G2 at T1 is consistent with the proposed film-forming potential of the biopolymer. Nevertheless, because the deposited layer was not directly visualized or characterized, film formation remains an interpretation supported by the combined skin biophysical responses.

TEWL represents the flux of water vapor through the stratum corneum and is widely used as a noninvasive indicator of skin barrier function.^48,49^ Higher TEWL values are generally associated with impaired barrier function, whereas lower values indicate reduced water loss through the skin.^48^

At T2, only the formulations containing the biopolymer, G2 and GC2, showed significant reductions in TEWL relative to their respective baseline values (*p* < 0.05), whereas no significant changes were observed for G1 and GC1 (*p* > 0.05) (Figure 6). This response is consistent with a contribution of the biopolymer to water retention at the skin surface.

**Figure 6:**
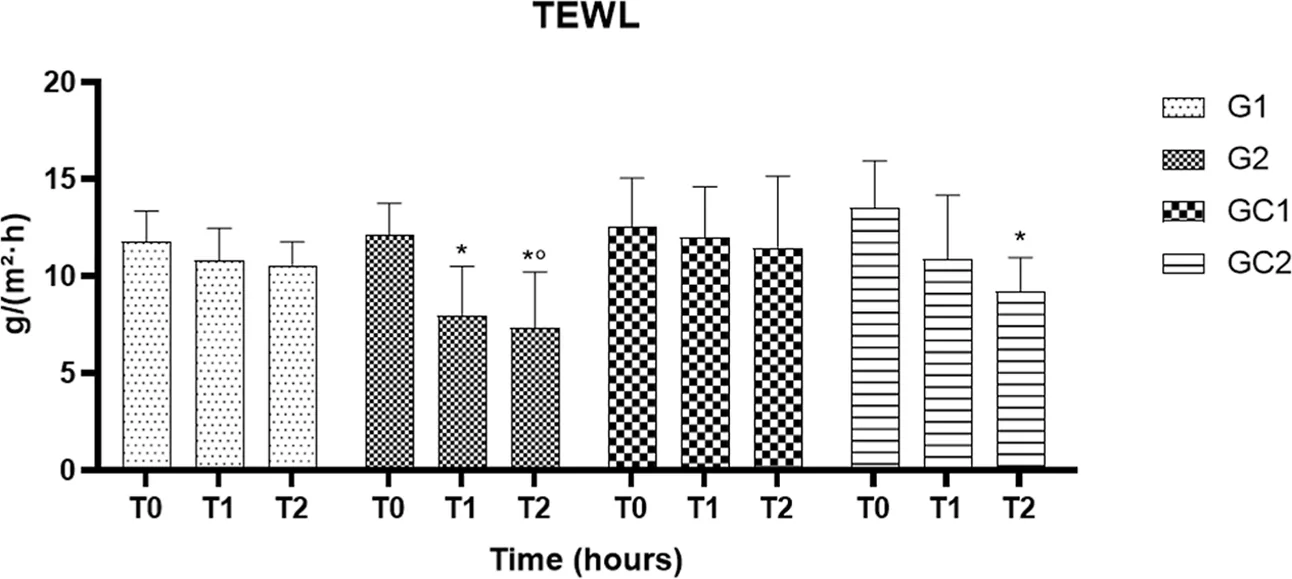
Transepidermal water loss before application (T0) and 1 h (T1) and 2 h (T2) after application of the gel (G1), gel containing 1% biopolymer (G2), gel-cream (GC1), and gel-cream containing 1% biopolymer (GC2). Data are presented as mean ± standard deviation, *n* = 8. *Significant difference from the respective baseline value (T0), *p* < 0.05. °Significant difference between G1 and G2 at T2, *p* < 0.05.

Film-forming polymers may reduce water evaporation by depositing a polymer-rich layer that produces a partial or semiocclusive effect on the skin surface. Previous studies with topical polymeric film-forming systems demonstrated that the nature of the formed film influenced TEWL and that semiocclusive films could significantly limit water evaporation^14,15,50^ Therefore, the reduction in TEWL observed for G2 and GC2 is consistent with the proposed film-forming potential of the tara/red algae biopolymer.

The TEWL reductions were accompanied by increases in stratum corneum water content, indicating complementary effects on water retention. G2 simultaneously increased stratum corneum water content and reduced TEWL at T1, whereas the significant TEWL response for GC2 became detectable at T2 (*p* < 0.05). This temporal pattern is consistent with an earlier barrier-related response for the biopolymer-containing gel under the evaluated conditions.

Moreover, G2 showed significantly lower TEWL than G1 at T2 (*p* < 0.05). This direct comparison supports the contribution of the biopolymer to the reduction in water loss when added to the gel formulation. The absence of a corresponding direct difference between GC1 and GC2 does not exclude a biopolymer contribution in the gel-cream, but indicates that the effect was more clearly demonstrated in the gel system under the evaluated conditions.

The earlier response of G2 may be associated with the composition and structural organization of the gel, which differs from the gel-cream in the absence of an oil phase and emulsifying system. However, as previously discussed, these formulation variables were not evaluated separately; therefore, the effect should be attributed to the gel as a whole rather than exclusively to the absence of oily components.

Overall, the significant reductions in TEWL observed for G2 and GC2 support a short-term barrier-related benefit of the biopolymer. The concurrent increase in stratum corneum water content and reduction in TEWL, particularly for G2 at T1, is consistent with the deposition of a hydrated polymer-rich layer that limits water evaporation. Because the morphology and continuity of this layer were not directly characterized, these findings support the film-forming potential of the biopolymer through complementary indirect evidence.

Regarding skin microrelief, the regions treated with G2 and GC2 showed significant improvements in all evaluated SELS parameters at T2 relative to their respective baseline values (*p* < 0.05) (Figures 7–9). Both formulations increased the skin smoothness index (SEsm) and reduced the skin roughness (SEr) and scaliness (SEsc) indices. In addition, SEsm was significantly higher in the region treated with GC2 than in that treated with GC1 at T2 (*p* < 0.05), supporting a contribution of the biopolymer to the skin-smoothing response of the gel-cream formulation (see Figure 8).

**Figure 7:**
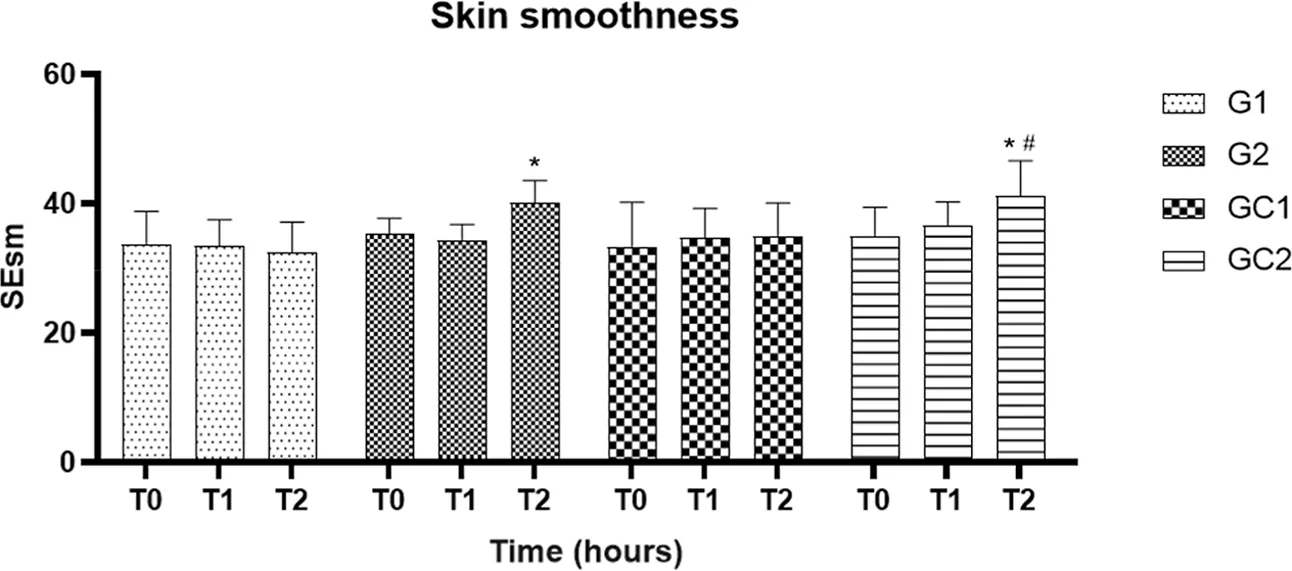
Skin smoothness before application (T0) and 1 h (T1) and 2 h (T2) after application of the gel (G1), gel containing 1% biopolymer (G2), gel-cream (GC1), and gel-cream containing 1% biopolymer (GC2). Data are presented as mean ± standard deviation, *n* = 8. *Significant difference from the respective baseline value (T0), *p* < 0.05. #Significant difference between GC1 and GC2 at T2, *p* < 0.05.

**Figure 8:**
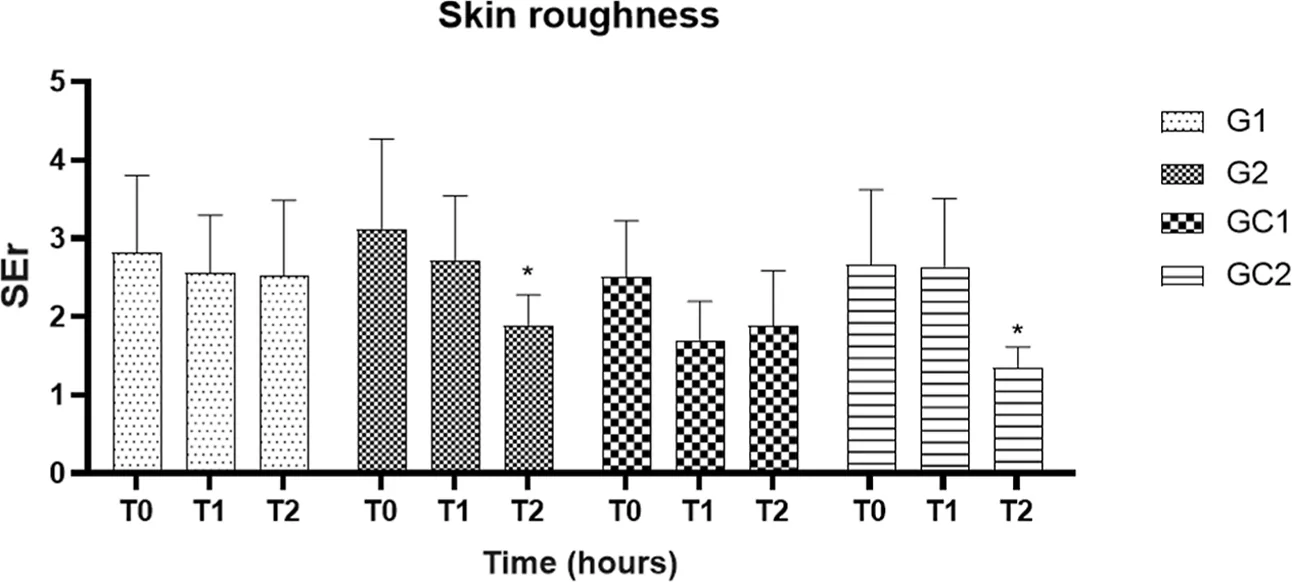
Skin roughness before application (T0) and 1 h (T1) and 2 h (T2) after application of the gel (G1), gel containing 1% biopolymer (G2), gel-cream (GC1), and gel-cream containing 1% biopolymer (GC2). Data are presented as mean ± standard deviation, *n* = 8. *Significant from the respective baseline value (T0), *p* < 0.05.

**Figure 9:**
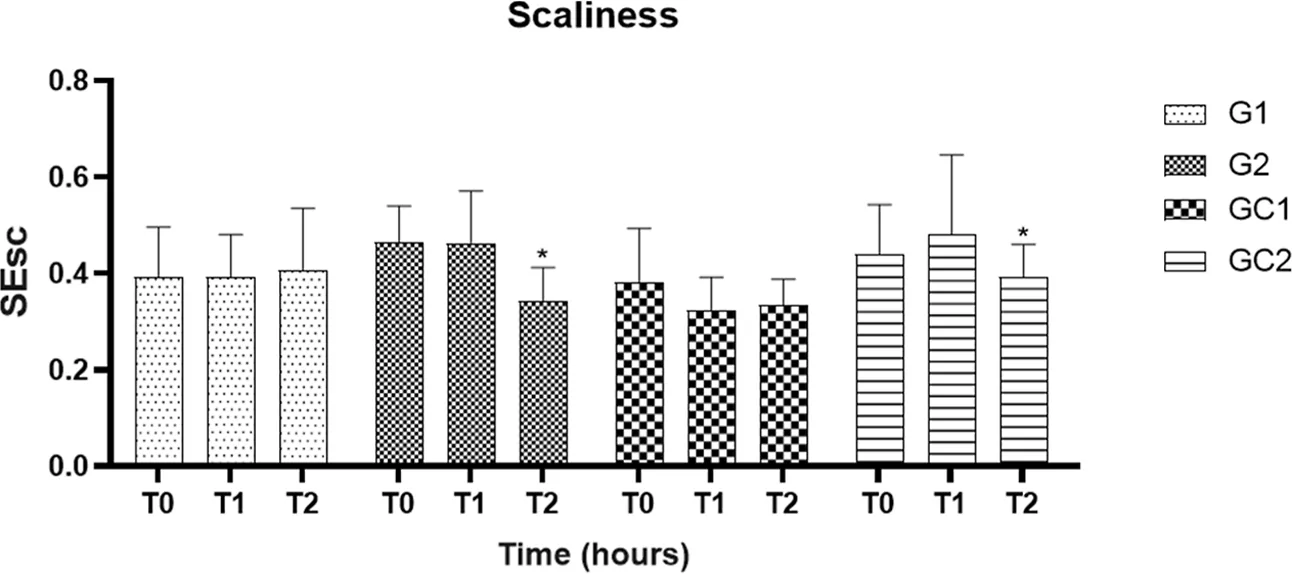
Skin scaliness before application (T0) and 1 h (T1) and 2 h (T2) after application of the gel (G1), gel containing 1% biopolymer (G2), gel-cream (GC1), and gel-cream containing 1% biopolymer (GC2). Data are presented as mean ± standard deviation, *n* = 8. *Significant from the respective baseline value (T0), *p* < 0.05.

In the SELS analysis used in the present study, increased SEsm values indicate improved skin smoothness, whereas reductions in SEr and SEsc indicate lower skin roughness and scaliness, respectively.^51–53^ Therefore, the combined changes in these parameters demonstrate an improvement in the skin surface profile after application of the biopolymer-containing formulations.

The microrelief responses were consistent with the increases in stratum corneum water content observed for G2 and GC2. Increased hydration may reduce the prominence of superficial irregularities and improve the smoothness and uniformity of the skin surface. Concurrent improvements in skin hydration and microrelief have also been reported after the application of cosmetic formulations containing polysaccharide-based ingredients.^52,54^

The hydrophilic character of the tara/red algae biopolymer may have contributed to water retention and the deposition of a hydrated polymer-rich layer on the skin surface. Such a layer may temporarily reduce the appearance of superficial irregularities, thereby contributing to increased smoothness and reduced roughness and scaliness. Thus, the combined SELS results are consistent with the proposed film-forming potential of the biopolymer. However, because the morphology and continuity of the deposited layer were not directly characterized, the microrelief changes should be interpreted as complementary indirect evidence supporting the proposed film-forming potential.^15,54^

Although both biopolymer containing formulations improved the skin surface profile at T2, the significant difference between GC2 and GC1 in SEsm parameter indicates a more clearly demonstrated vehicle-controlled effect on skin smoothness in the gel-cream system. This response may reflect the combined contribution of the biopolymer and the gel-cream. Formulation composition and emollient selection can influence the physical–mechanical properties and application performance of topical formulations.^46^ Emollient-containing formulations have also been reported to improve skin hydration^55^ and, depending on the complete formulation, skin microrelief.^56^ However, because the oil phase, emulsifying system, and other formulation components were not evaluated separately, their individual contributions cannot be distinguished.

The representative skin microrelief images shown in Figure 10 qualitatively illustrate the changes detected by the SELS parameters, including improved skin smoothness and reduced roughness and scaliness after application of G2 and GC2.

**Figure 10:**
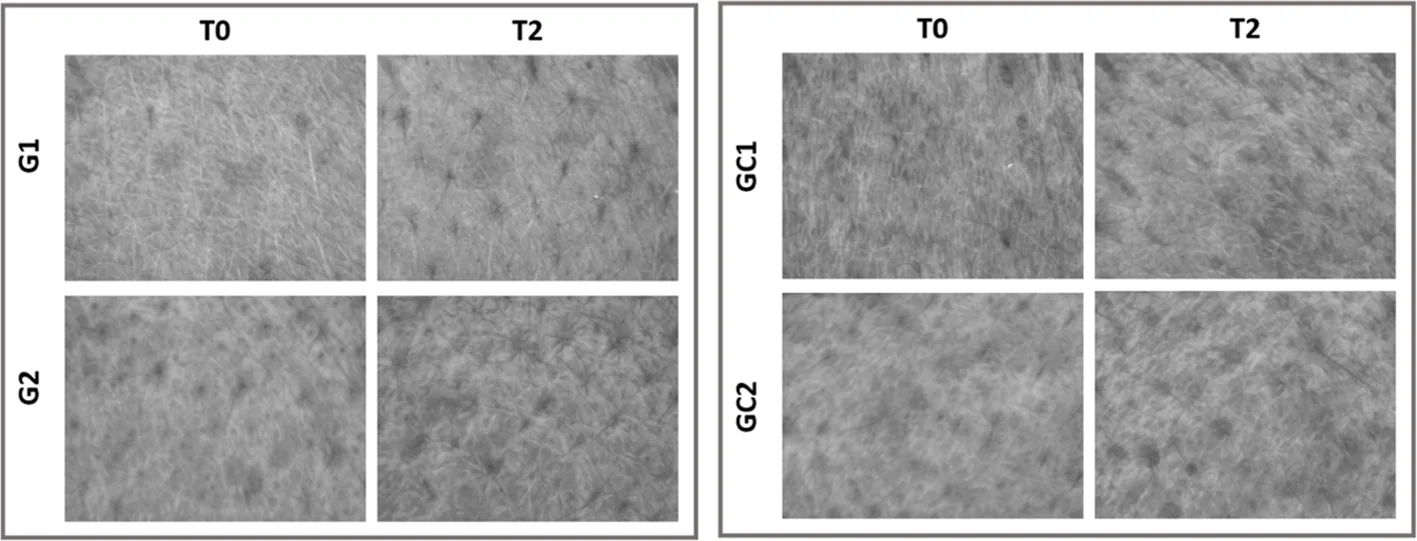
Representative Visioscan VC 98 images of the skin microrelief in the malar region before application (T0) and 2 h after application (T2) of the gel (G1), gel containing 1% biopolymer (G2), gel-cream (GC1), and gel-cream containing 1% biopolymer (GC2).

Overall, the gel formulation showed an earlier barrier-related response, whereas the gel-cream showed the clearest vehicle-controlled improvement in skin smoothness. In both formulations biopolymer addition modified the rheological and texture profiles without alteration evident on application-related sensory properties. The agreement between these formulation properties and the clinical responses supports the influence of formulation composition of the biopolymer performance. These findings demonstrate that the same biopolymer can produce distinct temporal and skin biophysical responses depending on the composition and structural organization of the formulation. The short-term clinical assessment involved eight participants and did not evaluate the persistence of the effects beyond 2 h, while film formation was inferred indirectly rather than directly characterized. Future studies using direct film-characterization techniques, longer evaluation periods, and pollutant-challenge models could further clarify the underlying mechanism and extend the present findings.

## 4 Conclusion

The tara and red algae biopolymer modified the rheological and textural properties of both gel and gel-cream formulations without alteration evident on application-related sensory properties. Formulations composition influenced the short-term clinical response: the gel formulation showed an earlier increase in stratum corneum water content and reduction in TEWL, whereas the gel-cream showed the clearest vehicle-controlled improvement in skin smoothness. Both formulations containing the biopolymer also improved skin roughness and scaliness parameters at T2. These findings demonstrate that the formulation composition influenced the timing and type of response associated with the biopolymer. The combined instrumental measurements and skin biophysical results support its proposed film-forming potential, although film formation was inferred indirectly. Direct film characterization and longer-term studies are recommended to confirm the proposed mechanism and persistence of the observed effects.
